# Experiences of mimicry in eating disorders

**DOI:** 10.1186/s40337-022-00607-9

**Published:** 2022-07-15

**Authors:** Savannah R. Erwin, Peggy J. Liu, Nandini Datta, Julia Nicholas, Alannah Rivera-Cancel, Mark Leary, Tanya L. Chartrand, Nancy L. Zucker

**Affiliations:** 1 Department of Psychology and Neuroscience, Duke University, 417 Chapel Drive, Durham, NC 27708 USA; 2 Fuqua School of Business, Duke University, Durham, NC USA; 3 Department of Psychiatry and Behavioral Sciences, Duke University School of Medicine, Durham, NC USA; 4 Present Address: Katz Graduate School of Business, University of Pittsburgh, Pittsburgh, PA USA; 5 Present Address: Department of Psychiatry and Behavioral Sciences, Stanford Medicine, Stanford, CA USA; 6 Present Address: Department of Psychological and Brain Sciences, University of Louisville, Louisville, KY USA

**Keywords:** Eating disorders, Imitative behavior, Social perception, Interpersonal relations, Nonverbal communication

## Abstract

**Background:**

People unknowingly mimic the behaviors of others, a process that results in feelings of affiliation. However, some individuals with eating disorders describe feeling “triggered” when mimicked. This study explores the effects of implicit non-verbal mimicry on individuals with a history of an eating disorder (ED-His) compared to healthy controls (HCs).

**Method:**

Women (*N* = 118, n_ED-His_ = 31; *M*_*age*_ = 21 years) participated in a laboratory task with a confederate trained to either discreetly mimic (Mimicry condition) or not mimic (No-Mimicry condition) the mannerisms of the participant. Participants rated the likability of the confederate and the smoothness of the interaction.

**Results:**

Participants in the No-Mimicry condition rated the confederate as significantly more likable than in the Mimicry condition, and ED-His rated the confederate as more likable than HCs. ED-His in the Mimicry condition rated the interaction as less smooth than HCs, whereas this pattern was not found in the No-Mimicry condition. Among ED-His, longer disorder duration (≥ 3.87 years) was associated with less liking of a confederate who mimicked and more liking of a confederate who did not mimic.

**Conclusions:**

We discuss the implications of these findings for interpersonal therapeutic processes and group treatment settings for eating disorders.

**Plain English summary:**

Our study on subtle, nonverbal mimicry revealed differences in social behavior for women with a history of an eating disorder compared to healthy women. For participants with an eating disorder history, a longer duration of illness was associated with a worse pattern of affiliation, reflected in lower liking of a mimicker. Further research on how diverging processes of affiliation may function to perpetuate the chronicity of eating disorders and implications for treatment is needed.

**Supplementary Information:**

The online version contains supplementary material available at 10.1186/s40337-022-00607-9.

## Background

In general, comparing oneself to others has been shown to increase disordered eating [1–4]. A compelling example of this occurs in online communities, such as “Pro-Ana” (Pro-Anorexia) communities. Within these communities, individuals share photos of the impact of dangerous dietary restriction and tips on enhancing harmful weight loss strategies, personal examples which may negatively influence vulnerable individuals who seek to copy these behaviors or emulate these ascribed values [5, 6]. Examples of harmful social influence may also extend to treatment contexts. Colton and Pistrang [7] suggest that comparison to others may compromise the effectiveness, and possibly the safety, of inpatient treatment milieus, a point emphasized by Vandereycken [8] in his discussion of social contagion in specialized eating disorder treatment units. Social contaigion in these settings often takes the form of patients learning additional disordered behaviors (e.g.*,* surreptitious methods for purging or hiding food). Despite compelling examples of problematic explicit mimicry, imitation has not been systematically studied with an eating disorder population [5–7].

One potentially fruitful area of investigation is how those with a history of an eating disorder respond to being mimicked by someone else. While the examples from social media and contagion in a treatment setting describe explicit, intentional processes of imitation, implicit processes of mimicry have been associated with pro-social behaviors and increased affiliation among typically developing individuals. Prior research on mimicry by Chartrand and Bargh [9] found that engaging in similar behavior creates feelings of empathy and rapport between interactants. Additional research by Maurer and Tindall [10] found that when counselors mimicked the body positions of their clients, the clients perceived a greater level of expressed empathy on the part of the counselor. However, processes of mimicry in people with eating disorders may be disrupted at a fundamental, implicit level.

Processes of imitation often demand both physical and emotional closeness, a degree of intimacy in relationships that has been reported as feared in individuals with eating disorders. Prior research has identified a negative correlation between eating and shape concerns and the degree of comfort and closeness experienced in interpersonal relationships [11–15]. People with bulimia nervosa have been reported to experience interpersonal difficulties characterized by distrust, negative inetractions, and conflict with others [16]. For individuals with restrictive eating behaviors, fear of intimacy may be characterized by avoidance of expressing feelings of personal significance to others [16]. This avoidance has been theorized to be driven, in part, by the fear of and discomfort with perceived loss of control, a fear which is often reported among people with eating disorders [17]. Compared to healthy controls and individuals with a diagnosis of either depression or an anxiety disorder, people with anorexia nervosa (AN) endorse stronger motivation to avoid dependency and lower strivings for intimacy [18]. As intimate interpersonal relationships necessarily involve some degree of dependence on another person, avoidance of relationships may feel safer, to a degree, because this avoidance diminishes the uncertainty of relying on another person. However, such perceived safety may be at a significant physical and emotional cost. As secure, nonfamilial relationships may promote recovery from the eating disorder [19, 20], it is important to identify barriers to developing intimate relationships.

Evidence of early disruptions in attachment may contribute to such reluctance towards intimacy [16]. More specifically, insecure attachment with early caregivers has been theorized to be associated with disruptions in the emergence of a sense of self and increased eating disorder symptoms [21]. In the context of an optimal secure attachment, a child expresses a need in a manner that the caregiver can reliably decipher. The caregivere deciphers, responds to, and attempts to satisfy that need—often after some trial and error as the caregiver and child are developing more reciprocal, responsive interactions. Possible consequences of such reciprocal and responsive interactions are that the child feels seen, safe, and satisfied [22, 23]. Thus, the child’s ability to communicate needs and to be soothed and the caregiver’s ability to decipher and respond to the child’s needs are but some of the components that contribute to the emergence of a secure attachment [24].

As a result of these responsive, interactive processes [25–27], several adaptive intrapersonal and interpersonal processes are thought to occur. First, one may develop a sense of self-awareness: an individual learns that certain sensations communicate specific needs that can be satisfied (e.g., hunger, need for sleep, emotional experiences). Second, others are often viewed as validating and trusted sources of needs gratification: others both validate an individual’s legitimate needs and engage in behaviors aimed to help with satisfying those needs. These processes are proposed to contribute to an individual’s sense of self-awareness and trust in others. However, these processes may become disrupted when one experiences attachment relationships as insecure: individuals may struggle to develop accurate attunement to their own needs and feel increasingly vulnerable and dependent on the responsiveness of others, yet find those responses unreliable and therefore confusing and potentially invalidating [28]. Thus, early experiences of insecure attachment may further contribute to the “push and pull” motivation of individuals with eating disorders to seek interpersonal closeness and then feel threatened by such closeness [29].

Without a foundation of sufficient, accurate, empathic responses from caregivers, one consequence is that people may feel dependent on and fearful of the perceptions of others [30]. This heightened fear of negative evaluation can contribute to hypervigilance to signals of rejection in social situations and interfere with the ability to establish relationships [31]. Interpersonal closeness has the potential to be experienced as a rewarding level of intimacy yet is equaly fraught with the looming threat of unreliable responsiveness and, ultimately, fears of abandonment [28]. Evidence of unhealthy attachments and challenges forming intimate friendships [16, 29, 32–34] provides the impetus for studying fundamental processes that may be disrupted in persons with eating disorders.

Finding strategies to increase intimacy in persons with eating disorders is essential for several reasons. All effective treatments to date for eating disorders are interpersonal [35, 36]. Yet, a significant minority of individuals with an eating disorder fail to present for treatment, terminate treatment prematurely, or fail to respond to our most effective interventions [37–39]. Additionally, people with lived experiences of eating disorders consistently report that recovery is largely influenced by a sense of connection to self and others [19, 20, 40–42]. Finding strategies that allow individuals with eating disorders to be comfortable with others may increase the effectiveness of existing interventions, increase willingness to seek help, and may provide alternative sources of reinforcement that may compete with the reinforcement of disordered eating, such as the drive for thinness that constitutes a core aspect of AN.

Disruptions in awareness and understanding of internal body sensations can, in turn, impact susceptibility to social influence [43]. An individual’s trust in the veracity of the messages their body communicates and in their own capacity to decipher and respond to those messages may be protective against a variety of negative social influences—thereby reducing the perceived threat of others [44].

The present study examined how adults with a history of an eating disorder (ED-His) reacted when they were being unconsciously mimicked by a study confederate relative to healthy controls. This confederate either maintained a neutral posture or subtly mirrored the participant’s postures, movements, and mannerisms. Because of the interpersonal challenges and putative social deficits that have been shown in people with histories of AN or bulimia nervosa (BN), we hypothesized that ED-His participants would rate the mimicking confederate less favorably (i.e., lower likability and smoothness) than HCs. In contrast, we hypothesized that body awareness would moderate the relationship between mimicry and affiliation ratings, such that positive body awareness would be associated with greater affiliation (i.e., higher likability, smoother interaction rating) of a mimicker.

Individual differences in eating disorder histories may also be important. Consistent with the literature on the effects of starvation on social isolation [45], within the ED-His group, we hypothesized that duration of illness would moderate the relationship between mimicry and social affiliation, such that longer durations of illness would be associated with decreased perceived likability and lower smoothness ratings of the confederate when mimicked.

Finally, challenges with interpersonal intimacy may be influenced by associated symptoms that have been reported in people with eating disorders. For instance, there has been increasing study of the presence and influence of symptoms or diagnoses of autism spectrum disorder in those with eating disorders [46]. Autism spectrum disorder (ASD) is a neurodevelopmental disorder in which deficits in social-emotional reciprocity, such as abnormal social approach, are core defining features. There have been numerous studies of the associations of mimicry, specifically spontaneous facial mimicry, with features of ASD. Across studies, findings show that individuals with elevated ASD symptoms engage in reduced spontaneous facial mimicry of others [47–49]. Futher, several studies have demonstrated an association between the degree of social reward valuation and the degree of mimcry suggesting that the degree of mimicry is modulated by the perceived value of the social object [50–53]. The experience of being mimicked by someone else has been less studied in ASD. In an investigation of the experience of facial mimicry, Hsu et al. [54]. examined reward-related activation in the ventral striatum to faces that had mimicked the facial affect of an individual with autism relative to controls. In this study, participants were instructed to display a certain form of facial affect (Happy vs Sad) and then immediately viwed a movie of an actor performing a congruent or non-congruent facial expression. Findings revelaed that individuals with ASD demonstrated reduced reward-related neural activation when shown faces of actors who had mimicked their facial affect. To our knowledge, implicit behavioral mimicry with a live actor has not ben investigated in individuals with ASD, thus we conducted sample-wide exploratory analyses on the relationship between autism spectrum symptomology and reactions to mimicry to investigate the degree to which these symptoms are associated with implicit processes of affiliation [55].

In summary, this study examined processes of implicit non-verbal mimicry in adults with a history of an eating disorder (ED-His). We hypothesized that ED-His participants would rate the mimicking confederate less favorably (i.e., lower likability and smoothness) than HCs, a pattern that would demonstrate an atypical response to affiliative responses from others even when these affiliative processes occur without awareness. We further hypothesized that body awareness would moderate the relationship between mimicry and affiliation ratings, such that positive body awareness would be associated with greater affiliation (i.e., higher likability, smoother interaction rating) of a mimicker. We also hypothesized that duration of illness would moderate the relationship between mimicry and social affiliation, such that longer durations of illness would be associated with decreased perceived likability and lower smoothness ratings of the confederate when mimicked. Combined this pattern of results would support decreased affiliative responses in response to implicit non-verbal mimicry in ED-His, a pattern that is proposed to strengthen as the duration of the eating disorder increases.

## Method

### Participants

We invited adult women (18–30 years old) from the community, and specifically people enrolled in the Duke Eating Disorder registry, to contact the research team about participating in our study. Interested participants completed a brief screen and an extensive electronic survey of eating disorder history to determine eligibility. An electronic copy of the written consent form was emailed to eligible participants. Participation in the Healthy Control group (HC) was restricted to people with no history of an eating disorder. Inclusion in the clinical group (ED-His) required a history of AN, subthreshold AN, bulimia nervosa, subthreshold bulimia nervosa, binge eating disorder, or eating disorder not otherwise specified (see Additional file 1), absence of current eating disorder threshold symptoms (i.e., binge eating, purging, laxative/diuretic use, driven exercise) for at least 6 months, and self-reported current BMI greater than or equal to 18.5. For this study we chose to recruit weight-restored individuals to better isolate the characteristics of this population from the effects of acute malnutrition.

### Materials

#### Screening questionnaire

Interested participants completed a survey to determine study eligibility and assess eating disorder history. This survey included basic demographics questions (i.e., age, gender, race/ethnicity), the Eating Disorders Examination Questionnaire (EDE-Q), and qualitative and quantitative questions about their eating disorder history.

##### Eating disorders examination questionnaire (EDE-Q)

The EDE-Q is a 28-item self-report questionnaire measuring eating disorder symptoms across four subscales: restraint, eating concern, shape concern, and weight concern. These scores are averaged to produce a global EDE-Q score. Higher scores on the EDE-Q indicate greater degree of eating disorder symptomology. In addition to subscale scores, the EDE-Q also measures frequency of eating disorder symptoms. The EDE-Q has been shown to be reliable and valid [56]. In this sample, all subscales showed acceptable internal consistencies; Cronbach’s alpha was 0.77, 0.76, 0.90, and 0.83 for the Restraint, Eating Concern, Shape Concern, and Weight Concern subscales, respectively.

##### Eating disorder history interview

Participants answered both qualitative and quantitative questions about their eating disorder history, including the course of each symptom (i.e., whether low weight was chronic or intermittent) modeled after items about disorder course from the Structured Interview of Anorexic and Bulimic Disorders [57]. Participants also reported age of onset, latency between onset and seeking treatment, and age at offset. Onset was determined based on response to: *At what age do you think you first developed an eating disorder?* And offset was determined based on response to: *At what age did you stop having an eating disorder for good?*

#### Post-lab survey

After the lab procedure, participants completed an electronic survey that asked self-report questions about their personality, body awareness, body shape and weight concerns, their experience of the lab task, and their impressions of the confederate. These questionnaires are described in greater detail below.

##### Body awareness questionnaire (BAQ)

Created by Shields et al. [58] the BAQ is an 18-item self-report measure of sensitivity to and trust in deciphering body signals. An example item is: “I can distinguish between tiredness because of hunger and tiredness due to lack of sleep.” Higher scores on the BAQ indicate greater sensitivity to and trust in the capacity to decipher somatic signals. In this sample, Cronbach’s alpha was 0.90 for the BAQ.

##### Body shape questionnaire (BSQ)

The BSQ is a 34-item self-report questionnaire measuring concern about body weight and shape [59]. Higher scores on the BSQ indicate greater concern about body weight and shape. The BSQ has been found to be a reliable and valid measure of body image in women [60]. In this sample, Cronbach’s alpha was 0.97 for the BSQ.

##### Autism-spectrum quotient (AQ)

The AQ is a 50-item self-report questionnaire that assesses a person’s endorsement of autism spectrum disorder symptomology based on the number of autistic traits they endorse across five domains: communication, social, imagination, local details, and attention switching. Higher scores on the AQ indicate greater endorsement of autism symptoms. AQ has shown high test–retest reliability and consistent agreement between self and parent report [55]. In this sample, Cronbach’s alpha was 0.73 for the AQ.

##### Affiliation ratings

Participants completed several ratings about their experience with the confederate, whom they were led to believe was another participant. For the two focal dependent variables, participants were asked to rate how likable they found the other participant (from 1 = *extremely unlikable* to 7 = *extremely likable*) and to respond to the prompt *how smoothly would you say your interaction went with the other participant?* also on a 7-point scale (1 = *extremely awkward* to 7 = *extremely smoothly*). These dependent variables, likable and smoothness ratings, were adapted from Chartrand and Bargh [9].

### Procedure

All study procedures were approved by the Duke University Institutional Review Board and conducted in accordance with the Declaration of Helsinki. Participation involved completion of an online screening survey and an in-lab portion in which participants were led to believe that they would be analyzing images with another participant. After completing the initial online screening survey, participants were invited to the lab for the experimental session. The lab portion incorporated a modified version of the procedure from Chartrand and Bargh’s Experiment 2 [9]. Participants from both groups (HC and ED-His) completed an “image analysis” task with a hypothesis-blind confederate who was acting as another participant. The advertised premise of the study was to develop an image set to be used for future personality assessment. The image analysis task consisted of the participant and confederate taking turns describing photos for 10–15 min.

Participants were randomly assigned to one of two conditions: Mimicry or No-Mimicry. In both conditions, the confederate refrained from smiling and making eye contact and maintained a neutral facial expression. In the Mimicry condition, the confederate mirrored the posture, movements, and mannerisms demonstrated by the participant. The confederate was trained to perform these movements subtly with the intention that the participant would have no awareness that she was being mimicked. This was intended to reflect the natural, unconscious processes of nonverbal imitation that occurs when people are interacting. In the No-Mimicry condition, the confederate maintained a neutral position with both feet on the floor and hands on the photos or in their lap. This neutral posture was established prior to initiation of the image analysis task.

After completion of the task, the confederate left the room, and the participant was asked to complete a questionnaire on the computer about their experience of the task, their impression of the other participant (the confederate), and their personality. A manipulation check was conducted at the end of the lab session: when the participant was done with the questionnaire, the researcher asked the participant *What do you think this study was about?* and recorded their response.

#### Data analysis

Data were analyzed using IBM SPSS Statistics Version 25.0. We used two-tailed independent *t-*tests with a Bonferroni correction to assess group differences in demographic characteristics. Differences between groups (HC and ED-His) and conditions (No-Mimicry and Mimicry) in likable and smoothness ratings were analyzed using two 2 × 2 ANOVAs. For effect size estimates, Cohen’s *d* was calculated for differences between cells of equal sizes and Hedge’s *g,* which uses a weighted pooled standard deviation, was calculated for differences between groups of unequal sizes. We also conducted linear regression analyses to test whether body awareness or duration of illness moderated the effects of mimicry on likable and smoothness ratings. Finally, we conducted two exploratory linear regressions to examine the relationship between autism symptoms, mimicry, and confederate ratings.

## Results

We first sought to characterize our sample by describing relevant demographic variables and eating disorder symptomology. Next, we reviewed data on the credibility of the experiment to assess whether participants detected the true nature of study. Primary analyses included two 2 × 2 ANOVAs with the main dependent variables: likable and smoothness ratings. Relevant individual differences (body awareness, duration of illness for ED-His group) were assessed for moderation. Finally, we conducted two exploratory simple regression analyses investigating the relationship between scores on the Autism Quotient and ratings of the confederate. These results are described in detail below.

### Sample characteristics

Our final sample consisted of 118 women (31 ED-His). The average age was 21.2 years (SD = 2.73), and the average education completed was 15.12 years (SD = 2.14). The majority of the sample identified as white (51.5%) or Asian (28.2%). A complete breakdown of the sample demographics by group and condition can be found in Table 1.

**Table 1:** Demographic characteristics by group and condition

Group	HC (n = 87)	ED-His (n = 31)		
**Eating disorder examination Questionnaire (EDE-Q)**				
Restraint*	1.24 (1.20)	2.31 (1.38)		
Eating concern*	0.59 (0.82)	1.41 (1.00)		
Shape concern*	1.37 (1.24)	2.84 (1.44)		
Weight concern*	1.14 (1.10)	2.44 (1.41)		
Global*	1.08 (0.95)	2.21 (1.15)		
Body shape questionnaire (BSQ)*	70.87 (20.16)	78.90 (16.64)		
Body awareness questionnaire (BAQ)*	74.92 (27.38)	115.07 (33.76)		
Autism quotient (AQ)	16.40 (5.62)	18.57 (6.03)		
Liking of confederate*	5.68 (0.87)	5.96 (0.86)		
Smoothness of interaction with confederate*	5.64 (1.15)	5.39 (1.33)		

Condition	No-mimicry	Mimicry	No-mimicry	Mimicry
Demographics	n (%)		n (%)	
**Race**				
White	16 (36.4%)	14 (32.6%)	13 (86.7%)	10 (62.5%)
African American	4 (9.1%)	4 (9.3%)	0 (0.0%)	1 (6.3%)
Hispanic/Latino	1 (2.3%)	3 (7.0%)	0 (0.0%)	0 (0.0%)
Asian	11 (25.0%)	12 (27.9%)	2 (13.3%)	4 (25.0%)
Indian	1 (2.3%)	2 (4.7%)	0 (0.0%)	0 (0.0%)
Pacific Islander	1 (2.3%)	0 (0.0%)	0 (0.0%)	0 (0.0%)
Native American	2 (4.5%)	0 (0.0%)	0 (0.0%)	0 (0.0%)
Missing	8 (18.2%)	8 (18.6%)	0 (0.0%)	1 (6.3%)

	Mean (SD)		Mean (SD)	
Age (years)	21.19 (2.64)	21.29 (3.07)	21.13 (2.29)	21.06 (2.77)
Education (years)	15.00 (2.20)	14.91 (2.09)	15.60 (2.10)	15.38 (2.28)
BMI	22.40 (3.62)	22.35 (2.91)	21.80 (2.24)	22.56 (2.21)

Group summaries are based on 31 participants with a prior diagnosis of Anorexia Nervosa (AN), Sub-AN, Bulimia Nervosa (BN), Binge Eating Disorder (BED), or Eating Disorder Not Otherwise Specified (EDNOS) and 87 sex, race, and age-matched healthy control participants. The groups (History of Eating Disorder (ED-His) and Healthy Control (HC)) and conditions (No-Mimicry and Mimicry) were not significantly different on any of the demographic variables. *Significant difference between group means at *p* < .05.

Data for all variables met the assumption of equal variances for independent-samples *t*-tests comparing the ED-His and HC groups. Participants in the ED-His group scored significantly higher than HCs on the EDE-Q Global scale, *t*(110) = 5.21, *p* < 0.001, *g* = 1.12; BSQ Total, *t*(116) = −6.58, *p* < 0.000, *g* = 1.38; and BAQ Total, *t*(116) = −1.99, *p* = 0.049, *g* = 0.420 (group means can be found in Table 1). Duration of illness reported by the ED-His group ranged from 1 to 10 years with an average of 3.69 years (SD = 2.42). Detailed information about the ED-His group and levels of symptomology can be found in Table 2.

**Table 2:** Characteristics of participants with a history of an eating disorder (ED-His)

Eating disorder characteristics	Mean (SD)
Age of onset (11–20)	15.17 (2.78)
Duration (years; 1–10)	3.69 (2.42)

	n (%)
**Diagnosis**	
AN	19 (61%)
Sub-AN	6 (19%)
BN	3 (10%)
BED	1 (3%)
EDNOS	2 (6%)
**Course**^**†**^	
Symptoms present ≤ 30% of the time	6 (19%)
Symptoms present 30–60% of the time	7 (23%)
Symptoms present 60–90% of the time	10 (32%)
Symptoms present 90–100% of the time	6 (19%)
**Treatment latency**^**‡**^	
Less than 6 months	1 (3%)
6–12 months	7 (23%)
13 months-3 years	6 (19%)
More than 3 years	1 (3%)
I never received treatment	14 (45%)

Symptom Abstinence^§^ (n (%))								
	Never did this	> 5 years	> 2 years	> 1 year	< 1 year	< 6 months	< 1 month	Still struggling
Binge Eating	9 (29%)	4 (13%)	2 (7%)	2 (7%)	1 (3%)	5 (16%)	5 (16%)	3 (10%)
Self-Induced Vomiting	14 (45%)	2 (7%)	3 (10%)	1 (3%)	2 (7%)	4 (13%)	4 (13%)	1 (3%)
Unhealthy Low Weight	13 (42%)	7 (23%)	4 (13%)	5 (16%)	2 (7%)	0 (0%)	0 (0%)	0 (0%)
Driven Exercise	2 (7%)	3 (10%)	7 (23%)	6 (19%)	4 (13%)	0 (0%)	1 (3%)	8 (26%)
Laxative Abuse	24 (77%)	2 (7%)	1 (3%)	0 (0%)	2 (7%)	0 (0%)	0 (0%)	2 (7%)
Diet Pill Abuse	24 (77%)	1 (3%)	0 (0%)	0 (0%)	1 (3%)	3 (10%)	0 (0%)	2 (7%)
Diuretic Abuse	29 (94%)	1 (3%)	0 (0%)	0 (0%)	0 (0%)	0 (0%)	0 (0%)	1 (3%)
Steroid Abuse	30 (97%)	1 (3%)	0 (0%)	0 (0%)	0 (0%)	0 (0%)	0 (0%)	0 (0%)
Food Restriction	15 (48%)	5 (16%)	1 (3%)	2 (7%)	1 (3%)	0 (0%)	1 (3%)	5 (16%)

^†^Illness course ratings were based on the time between first onset of symptoms and when participants considered themselves to have “stopped having an eating disorder.”

^‡^Treatment Latency was defined as time between onset of symptoms and initiation of treatment.

^§^Symptom Abstinence was in response to the question: “From today backwards, how long has it been since you engaged in the following symptom for at least a few times a week?”

### Manipulation check: ensuring participants were unaware of being mimicked

None of the participants conveyed awareness that the study was about perceptions of mimicry. One participant guessed that the study was about how the other participant’s movements affected her actions; however, this participant did not detect that she had been mimicked. Four participants accurately identified the other participant as a confederate but did not accurately identify the true nature of the study. Seven participants remarked that they knew the confederate prior to the study, but none of these seven detected that this “other participant” was acting as a confederate.

### Effects of condition and/or group membership on likability ratings

**Fig. 1:**
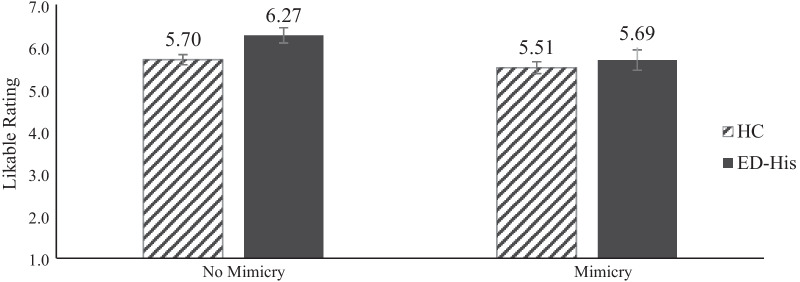
Participants rated how likable they found the confederate on a scale of 1 (extremely dislikable) to 7 (extremely likable). Mean likable rating was significantly higher for participants with a history of an eating disorder (ED-His) than for the healthy control (HC) participants and significantly higher for all participants in the No-Mimicry condition than for all participants in the Mimicry Condition. We did not find a significant group by condition interaction. Error bars represent standard error

Means for outcome variables are reported in Table 1. As shown in Fig. 1, the 2 × 2 ANOVA revealed a main effect of condition for likable ratings, such that averaging across groups, participants who were mimicked rated the confederate as less likable than participants who were not mimicked, *F*(1,114) = 3.40, *p* = 0.034, η_p_^2^ = 0.039 (see Additional file 1 for violin plots of these data). We also found a significant main effect of group, such that ED-His participants rated the confederate as more likable than HCs, *F*(1,114) = 2.80, *p* = 0.043, η_p_^2^ = 0.032. We did not find a significant group by condition interaction, *F(*1,114) = 1.18, *p* = 0.28, η_p_^2^ = 0.010.

### Effects of condition and/or group membership on ratings of smoothness

**Fig. 2:**
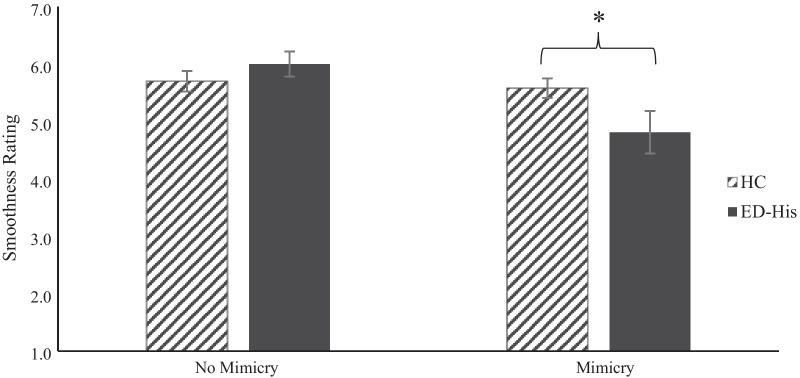
Participants rated the smoothness of the interaction with the confederate on a scale of 1 (extremely awkward) to 7 (extremely smoothly). Within the Mimicry condition, participants with a history of an eating disorder (ED-His) rated the interaction as less smooth than healthy control (HC) participants. There was not a significant group difference within the No-Mimicry condition. Error bars represent standard error

Our second 2 × 2 ANOVA with smoothness rating as the dependent variable showed that, averaging across conditions, there was not a significant difference between ED-His’s and HC’s ratings of the smoothness of the interaction, *F*(1,114) = 1.25, *p* = 0.27, η_p_^2^ = 0.011. We did find a main effect for condition, such that participants in the No-Mimicry condition rated the interaction as smoother than participants in the Mimicry condition, *F*(1,114) = 6.59, *p* = 0.012, η_p_^2^ = 0.055; however, this effect was qualified by a significant group by condition interaction, *F*(1,114) = 4.27, *p* = 0.041, η_p_^2^ = 0.036. Participants with a history of an eating disorder who were in the Mimicry condition rated the interaction as less smooth than participants who did not have a history of an eating disorder, *t*(56) = 2.12, *p* = 0.039, *g* = 0.62. There was no such difference between groups for the No-Mimicry condition, *t*(58) = 0.716, *p* = 0.48. These results are depicted in Fig. 2 (see Additional file 1 for violin plots of these data).

#### Exploration of individual differences

##### Body awareness

As measured by the BAQ, for which higher scores indicate more body-focused vigilance and greater ability to decipher the body’s signals, positive body awareness was not significantly correlated with likable ratings or smoothness ratings. The BSQ, for which higher scores indicate greater concern over body image and shape, negative body awareness was also not significantly correlated with confederate ratings.

##### Duration of illness

**Fig. 3:**
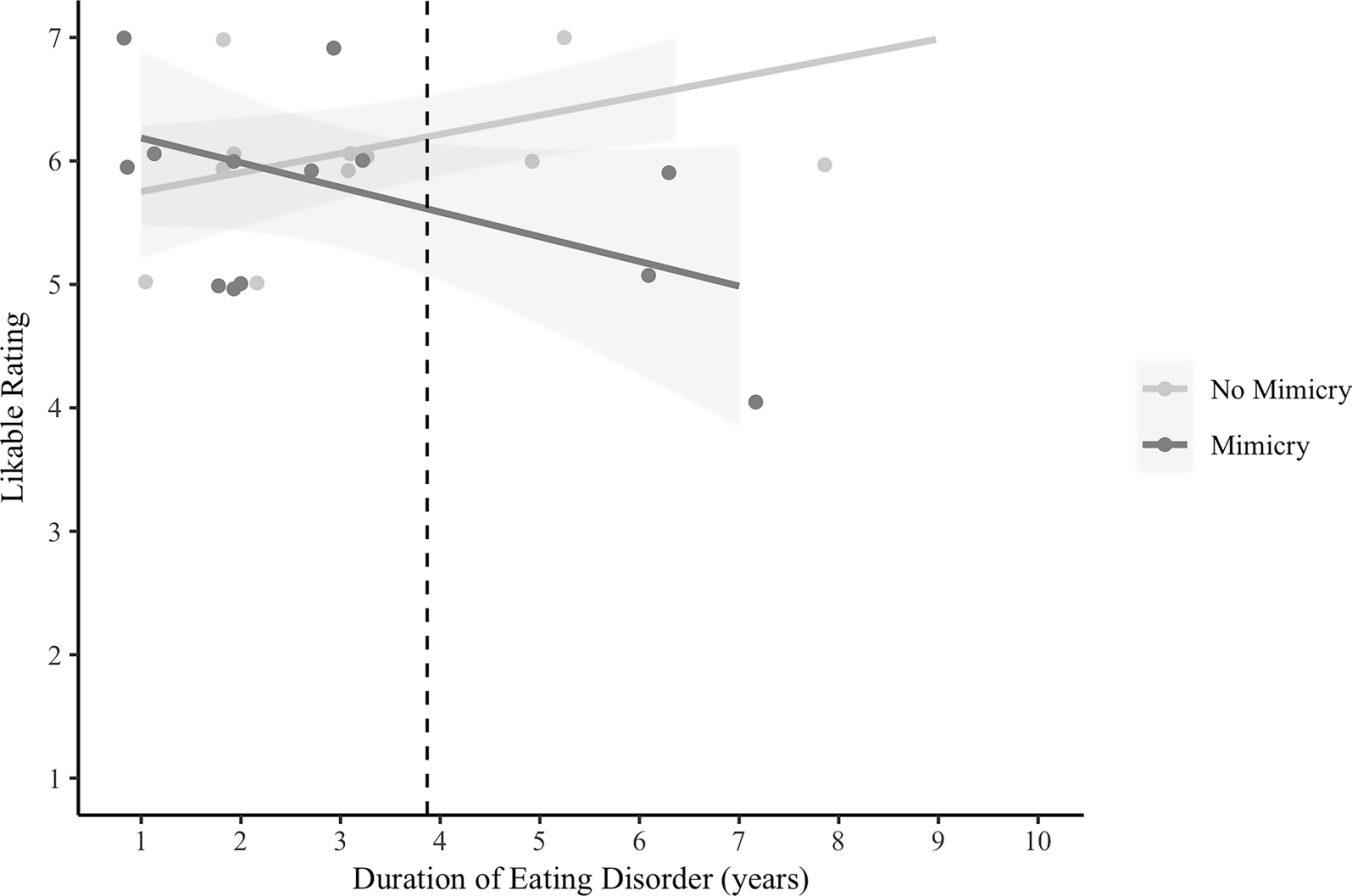
For participants with a history of eating disorder, self-reported duration of eating disorder moderated the effect of condition on likable ratings. Duration of illness showed a positive relationship with likable ratings in the No-Mimicry condition but showed a negative relationship with likable ratings in the Mimicry condition. The Johnson-Neyman Floodlight technique showed that the simple effect of condition on likable ratings is significant when the duration of eating disorder was 3.87 years or longer. The vertical dashed line indicates the region of significance for the simple effect within the ED-His group: for participants who had a duration of eating disorder of 3.87 years or longer, likeable ratings from participants who were not mimicked were significantly higher than those from participants who were mimicked. Scatterplot points are jittered to avoid overlap

Within the ED-His group, 29 individuals completed questions about how long they had experienced their eating disorder (duration of illness). Within the ED-His group, duration of illness was found to moderate the effect of mimicry on likable ratings of the confederate, *F*(1,25) = 3.95, *p* = 0.02, *R*^2^ = 0.32 (Fig. 3). Analysis of the simple slopes showed a positive relationship between duration and likable ratings in the No-Mimicry condition, such that a one-year increase in duration predicted a 0.15 unit increase in likable rating. By contrast, participants in the Mimicry condition showed the opposite pattern: a one-year increase in duration predicted a 0.20 unit decrease in likable rating. Examined differently, the Johnson-Neyman Floodlight technique showed that the simple effect of condition on likable ratings was significant when the duration of eating disorder was 3.87 years or longer [61]. We did not find that duration of illness significantly moderated the effect of mimicry condition on smoothness ratings, *F*(1,25) = 2.24, *p* = 0.11, *R*^2^ = 0.21.

#### Exploratory analyses

The AQ was also found to be significantly related to both dependent variables. Participants in the ED-His and HC groups were not significantly different on AQ. Linear regression models including AQ score and condition predicted a significant proportion of the variation in smoothness rating (*F* (1,108) = 3.15, *p* = 0.028, Adj. *R*^2^ = 0.0804) and likability (*F* (1,108) = 4.161, *p* = 0.007, Adj. *R*^2^ = 0.104). Within the Mimicry condition, a 1-unit increase in AQ score predicted a 0.06 unit decrease in smoothness rating (*t*(56) = −2.852, *p* = 0.006, *b* = −0.06) and a 0.07 unit decrease in likable rating (*t*(56) = −2.36, *p* = 0.022, *b* = −0.07).

## Discussion

This study investigated the experience of implicit nonverbal mimicry in a sample of women with a history of an eating disorder compared to healthy controls. Consistent with previous literature on mimicry, ratings of the likability of the confederate and smoothness of the interaction with the confederate were the main dependent variables of interest [18]. Contrary to our primary hypothesis, we found that across all groups participants who were not mimicked rated the confederate as more likable and the interaction as smoother than did participants who were mimicked. There is a large body of research supporting that subtle mimicry increases liking [9, 62–65]. Notwithstanding, the potential prosocial influences of mimicry have been reported to be minimized by certain interpersonal factors. Examples of interpersonal factors that decrease the prosocial influences of mimicry include when interaction partners are competing [66] or by the degree of perceived contingency of the mimicker’s action (whether the subject perceived they positively influenced the confederate’s actions) [67]. While we did not assess perceived competition of influence in this study, such assessment would strengthen the interpretations from this task in future research.

Whereas prior studies have found that being subtly mimicked increases the desire to affiliate with the mimicker [9, 62], our ED-His participants whose posture and mannerisms were subtly mirrored by a confederate rated the interaction as less smooth compared to ED-His participants who were not mimicked. This pattern of results suggests a disruption in the typical pattern of affiliation when someone is implicitly mimicked for people with a history of an eating disorder. Prior research has shown that, compared to healthy individuals, people who have a history of an eating disorder are subconsciously more vigilant of others’ bodies [68]. It could follow that our ED-His participants may have been more sensitive to the mimicry due to their hypervigilance of others’ bodies. While the manipulation was successful, it is possible that attentiveness towards others contributed to an impression of reduced smoothness.

An alternative explanation is that this difference between conditions reflected negative self-judgments in the context of an interpersonal interaction. Individuals with eating disorders have been reported to become fixated on social cues of rejection and are more avoidant of social cues of reward [69]. Unlike likability ratings, ratings of smoothness reflect judgments of the dyad—how two people interact with each other. Given fears of intimacy and negative evaluation cited earlier, it is possible that the experience of nonverbal mimicry is threatening due to potential rejection, contributing to more scrutiny of oneself in an interaction. Alternatively, prior research has shown that perceived social competition can modulate mimicry and although our participants were instructed to work collaboratively with the confederate, the tendency for individuals with eating disorders to experience others as competitors could have negatively affected their valuation of mimicry [16, 66].

Prior research suggests that individuals with eating disorders experience conflicting and ambivalent motivations towards relationships and this may make potentially close relationships the subject of scrutiny [16, 29]. Given that the current study attempted to manipulate more implicit processes, it is notable that these potentially protective and defensive responses may have been elicited in this context. Because our ED-His participants were all weight-restored (BMI ≥ 18.5), this difference may reflect a characteristic deficit in social affiliation for people with an eating disorder or a scar from the acute stage of the eating disorder.

Implicit awareness of the relative congruency between one’s own and another’s body movements may be important for experiencing mimicry as prosocial and may be predicated on body awareness. Thus, we sought to better characterize the relationship between mimicry and social affiliation by investigating the impact of positive and negative body awareness on ratings of the confederate. Our failure to find a significant association may indicate that an alternative facet of interoception is more crucial to experiences of affiliation. There has been increased precision in operationalizing component of interoception, including processes of awareness and accuracy, such as those subjectively captured by the Body Awareness Scale, but also more metacognitive evaluations of these perceptions [70]. For example, the construct of ‘body trust’ has been increasingly operationalized and investigated in those with eating disorders [71]. Body trust is the degree to which sensations perceived in the body are viewed as accurate and worthy of responsive actions [72]. It could be that facets such as body trust or rmore direct measures of how the individual attunes to perceived body sensations are more relevant for affiliation than body awareness. This would be theoretically consistent with findings from the attachment literature which show that responsive interactions are crucial for secure attachment. Perhaps accurate self-attunment, or ‘self-parenting,’ may be a crucial variable in experiencing safety with another and valuing interpersonal closeness.

In order to expand on our finding that ED-His participants did not respond in an affiliative manner to a mimicking confederate, we conducted exploratory analyses. Specifically, the Autism Spectrum Quotient (AQ) total score was explored as a possible moderator of the relationship between mimicry and confederate ratings. Symptoms or diagnosis of autism in people with eating disorders have been reported to influence treatment and potentially prolong disorder course [73]. Challenges with social reciprocity, including emotion perception and expression, have been documented, which are compontents of social affiliation that may impact the development of intimate relationships. While deficits in social reciprocity are indexed by the AQ [55], our ED-His group did nto score significantly higher than healthy controls. Thus, we investigated this moderation across the entire sample. AQ total score showed a significant negative correlation with both likable and smoothness ratings. This is consistent with prior research that has found lower reward-related neural responses to mimicry in individuals with ASD compared to neurotypical individuals [54]. In this study, one hypothesis is that individuals with higher autistic features did not experience mimicry as reward as healthy controls, as evidnced by lower ventrial striatum activity in response to mimicry. This differential neural activity may reflect lower interest in and pleasure from social engagement, patterns of valuation which may contribute to social avoidance and influence social reciprocity in individuals on the autism spectrum [54, 74]. We also found a stronger negative relationship between smoothness ratings and AQ total score for the Mimicry condition than the No-Mimicry condition. Taken together, our data suggest that people who endorse greater difficulties with social reciprocity may perceive a mimicker as less warm and the interaction as less smooth than with a non-mimicking interaction partner. Future research could explore the potential for differing reactions across other types and degrees of mimicry.

We also looked at the effect of duration of illness on the regression of likable ratings on mimicry (vs. control) condition. We found that, within the ED-His group, self-reported duration of illness moderated the relationship between mimicry and likable rating such that a longer duration of eating disorder was associated with a worse pattern of affiliation, reflected in lower liking of a mimicker. While prior research shows that mimicry can facilitate affiliative processes [9, 75], our study suggests that women with a history of an eating disorder experience mimicry as distancing, and the longer her history of eating disorder, the less affiliation she will feel towards a mimicker. Explicit behaviors such as avoiding social engagements as a means to escape uncomfortable eating situations [76], rigid routines that interfere with spontaneous social interactions [77], and social cognitive deficits that may contribute to social relations being more difficult or nonrewarding have been suggested [77, 78]. Findings from the current study provide a potential contribution to this characterization of isolation: implicit processes of mimicry may become more disrupted as the disorder progresses. In AN, the acute state of the disorder is a state of threat: the human being is starved. Perhaps primal instincts motivating distance from predators or from conspecifics that would share one’s food become activated and habitual as the disorder progresses. Additionally, this perception of mimicry as less prosocial could reflect increased hypervigilance of others’ bodies and greater bias towards social rejection for women with longer disorder histories. Given the importance of interpersonal connectedness in promoting recovery from an eating disorder [20], and the prevalence of implicit, unintentional mimicry in healthy, secure relationships [62, 79, 80], finding ways to maximize the benfits of mimicry in this population is of critical importance.

Current findings hold possible treatment implications: mimicry can bee a strong therapeutic tool. Across therapeutic modalities, subtle mimicry (e.g., body posture, hand movements, vocal pitch) has been shown to positively impact patients’s feelings about the therapeutic alliance, increase how empathic the therapist is perceived to be, and increase treatment effectiveness [81]. However, given our data suggest that mimicry is not perceived as positively by women with a history of eating disorder compared to healthy controls, mimicking a patient with a history of or an active eating disorder may not have the desired outcome of improving the therapeutic relationship. Keeping in mind that attempts to affiliate with patients, particularly those with a longer duration of illness, may negatively affect therapeutic alliance, we can develop strategies so that our approach behaviors are more cautious, which may have the ironic consequence of increasing likability. These results may also inform the implementation of group treatment models; specifically, to consider duration of illness when gradually titrating the degree of interaction with patients so as not to inadvertently motivate social distancing. There is further promise of the use of mimicry in therapy: interventions incorporating postural and facial mimicry have been shown to improve social skills, particularly emotion inference, in adults with ASD and social anxiety disorder [82, 83]. Application of these types of interventions in eating disorder treatment settings should be explored.

These conclusions are drawn with several limitations in mind which could be addressed in future studies. First, we did not collect judgments of how well the confederates engaged in mimicry of the participants. Our failure to replicate the key finding from Chartrand and Bargh [9] that healthy participants respond favorably to implicit nonverbal mimicry raises a question about how reliably our confederates mimicked the participants. Given that none of the participants recognized that they were being mimicked and that there were significant effects of group, it is possible that our confederates were not mimicking at the minimum threshold needed to influence perception of healthy individuals or that the manipulation was weaker than in prior studies. Despite obtaining a small effect of mimicry on smoothness ratings for ED-His, our manipulation may not have been strong enough to influence HCs. However, we did find significant differences between the Mimicry and No-Mimicry conditions, suggesting that there was enough mimicry by the confederates to elicit a reaction from some of our participants. Second, our small sample size of ED-His participants may have limited our ability to detect the hypothesized interaction effects. Future studies should recruit larger samples of ED-His participants to test if the initial patterns revealed by our exploratory analyses hold. Lastly, the present study relied on self-reported information of eating disorder histories and behaviors. Although self-report via electronic survey can facilitate honesty and openness in response to sensitive questions, we are unable to confirm the accuracy of self-described eating disorder history. Given the discrepancies in reporting that we were able to identify, it is possible that there were other inaccuracies that we were not able to detect and that may have influenced our results. Future studies may consider looking further into the intensity and impairment associated with pre-illness anxiety and poor relationship with parents throughout childhood as potential confounders of treatment history.

## Conclusions

In summary, our findings show that relative to healthy controls, women with a history of an eating disorder who were presently weight restored demonstrated differences in their unconscious perception of mimicry, which is fundamental for affiliation. It is important to understand how these diverging processes of affiliation may function to perpetuate a chronic course of illness in eating disorders.

## Supplementary Information

**Additional file 1**. Diagnostic Criteria for Clinical (ED-His) Group; Violin Plots for Likable Ratings; Violin Plots for Smoothness Ratings. Description of data: a table including detailed descriptions of the diagnostic criteria used to determine inclusion in clinical group for our study and two violin plots displaying distributions of the primary dependent variables across groups.

## Data Availability

The dataset generated and analyzed for this study are available on the Open Science Framework: https://osf.io/vh4gy/?view_only=c585aaef76344cd29f147e10b30fbce3.

## Abbreviations

ED: Eating disorders
HC: Healthy controls
ED-His: Individuals with a history of an eating disorder
AN: Anorexia nervosa
BN: Bulimia nervosa
ASD: Autism spectrum disorders
BMI: Body mass index
EDE-Q: Eating Disorders Examination Questionnaire
BAQ: Body Awareness Questionnaire
BSQ: Body Shape Questionnaire
AQ: Autism-Spectrum Quotient
ANOVA: Analysis of variance
