# Supplementary material for: Experiences of mimicry in eating disorders
Source: J Eat Disord. 2022 Jul 15;10:103. doi: 10.1186/s40337-022-00607-9 (PMC9288029; doi:10.1186/s40337-022-00607-9)
Supplement: Supplementary file 1 — Additional file 1. Diagnostic Criteria for Clinical (ED-His) Group; Violin Plots for Likable Ratings; Violin Plots for Smoothness Ratings. Description of data: a table including detailed descriptions of the diagnostic criteria used to determine inclusion in clinical group for our study and two violin plots displaying distributions of the primary dependent variables across groups. [file 40337_2022_607_MOESM1_ESM.docx]

*Diagnostic Criteria for Clinical (ED-His) Group*

| Diagnosis | Criteria |
| --- | --- |
| Anorexia Nervosa (AN) | 1. Refusal to maintain body weight at or above a minimally normal weight for age and height 2. Intense fear of gaining weight or becoming fat 3. Disturbance in the way in which one’s body weight or shape is experienced, undue influence of body weight or shape on self-evaluation, or denial of the seriousness of the current low body weight 4. Amenorrhea (absence of at least three consecutive menstrual cycles) |
| Subthreshold Anorexia Nervosa (Sub-AN) | AN criteria B-D, with lowest BMI 17.6-18.5 OR AN criteria A-C, without amenorrhea OR AN criteria A and D, but no body image disturbance or intense fear of becoming fat |
| Bulimia Nervosa (BN) | 1. Recurrent episodes of binge eating, characterized by (1) eating, in a discrete period of time, an amount of food that is larger than what most people would eat and (2) a sense of lack of control overeating during the episode 2. Recurrent inappropriate compensatory behavior in order to prevent weight gain 3. The binge eating and compensatory behaviors occur, on average, at least twice a week for at least 3 months 4. Self-evaluation is unduly influenced by body weight and shape 5. The disturbance does not occur exclusively during episodes of Anorexia nervosa |
| Binge Eating Disorder (BED) | 1. Recurrent episodes of binge eating, characterized by (1) eating in a discrete period of time, an amount of food that is larger than what most people would eat under similar circumstances and (2) a sense of lack of control overeating during the episode 2. The binge eating episodes are associated with at least three of the following: eating more rapidly than normal, eating until uncomfortably full, eating large amounts of food in the absence of hunger, eating alone because of embarrassment about quantity consumed, feeling disgusted with oneself, depressed, or very guilty after overeating 3. Marked distress regarding binge eating 4. The binge-eating occurs, on average, at least once a week for 3 months 5. The binge eating is not associated with regular use of inappropriate compensatory behaviors |
| Eating Disorder Not Otherwise Specified (EDNOS) | Symptoms characteristic of an eating disorder are present and cause clinically significant distress or impairment but do not meet the full criteria for a specific eating disorder |

*Note*. Diagnostic criteria are based on the American Psychiatric Associations’ Diagnostic and Statistical Manual of Mental Disorders, Fourth Edition (DSM-IV).

*Violin Plots for Likable Ratings*

*
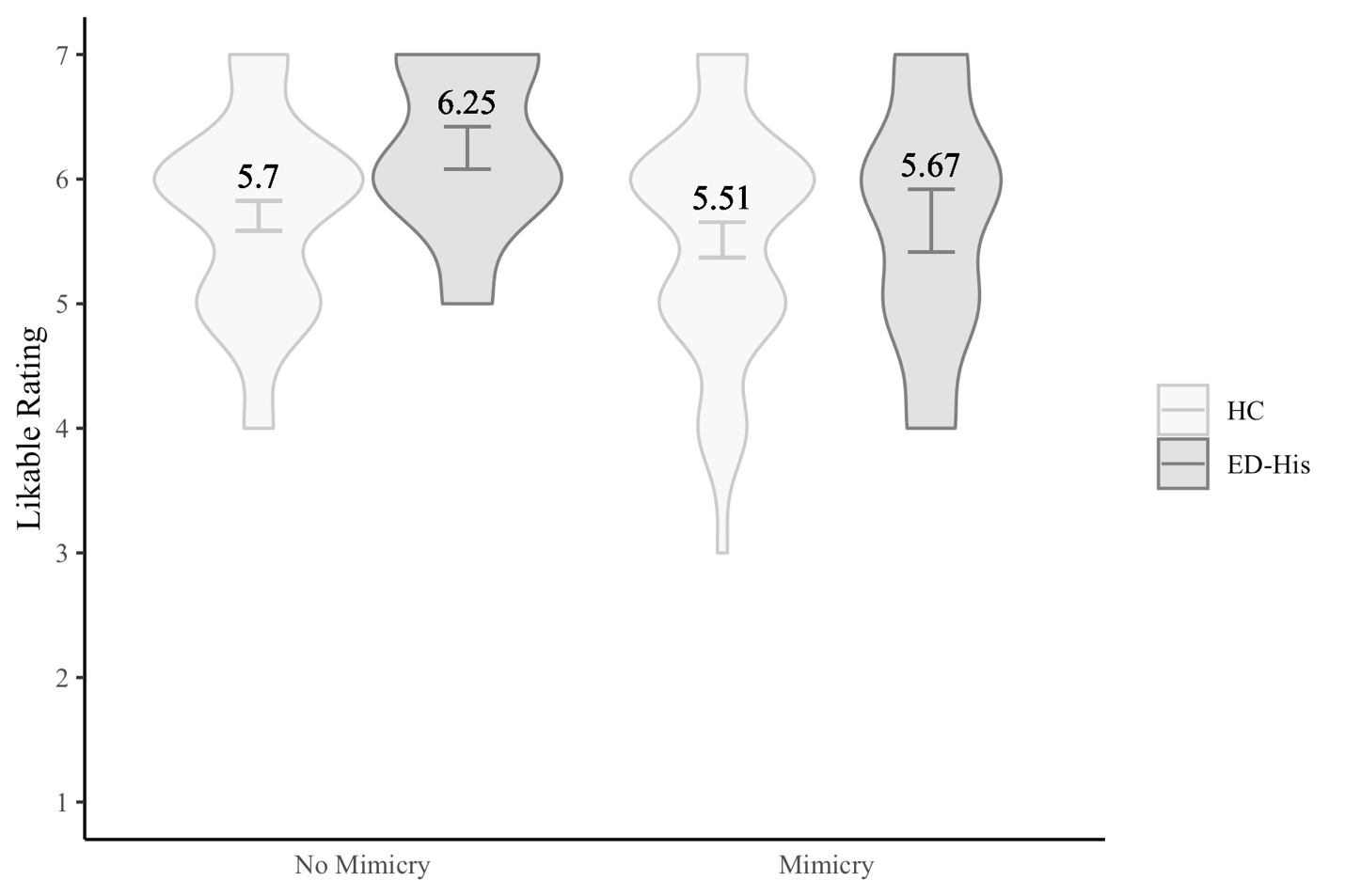
*

*Note*. Violin plots display the distribution of likable ratings. Participants rated how likable they found the confederate on a scale of 1 (extremely dislikable) to 7 (extremely likable). Mean likable rating was significantly higher for participants with a history of an eating disorder (ED-His) than for the healthy control (HC) participants and significantly higher for all participants in the No-Mimicry condition than for all participants in the Mimicry Condition. We did not find a significant group by condition interaction. Error bars represent standard error.

*Violin Plots of Smoothness Ratings*

*


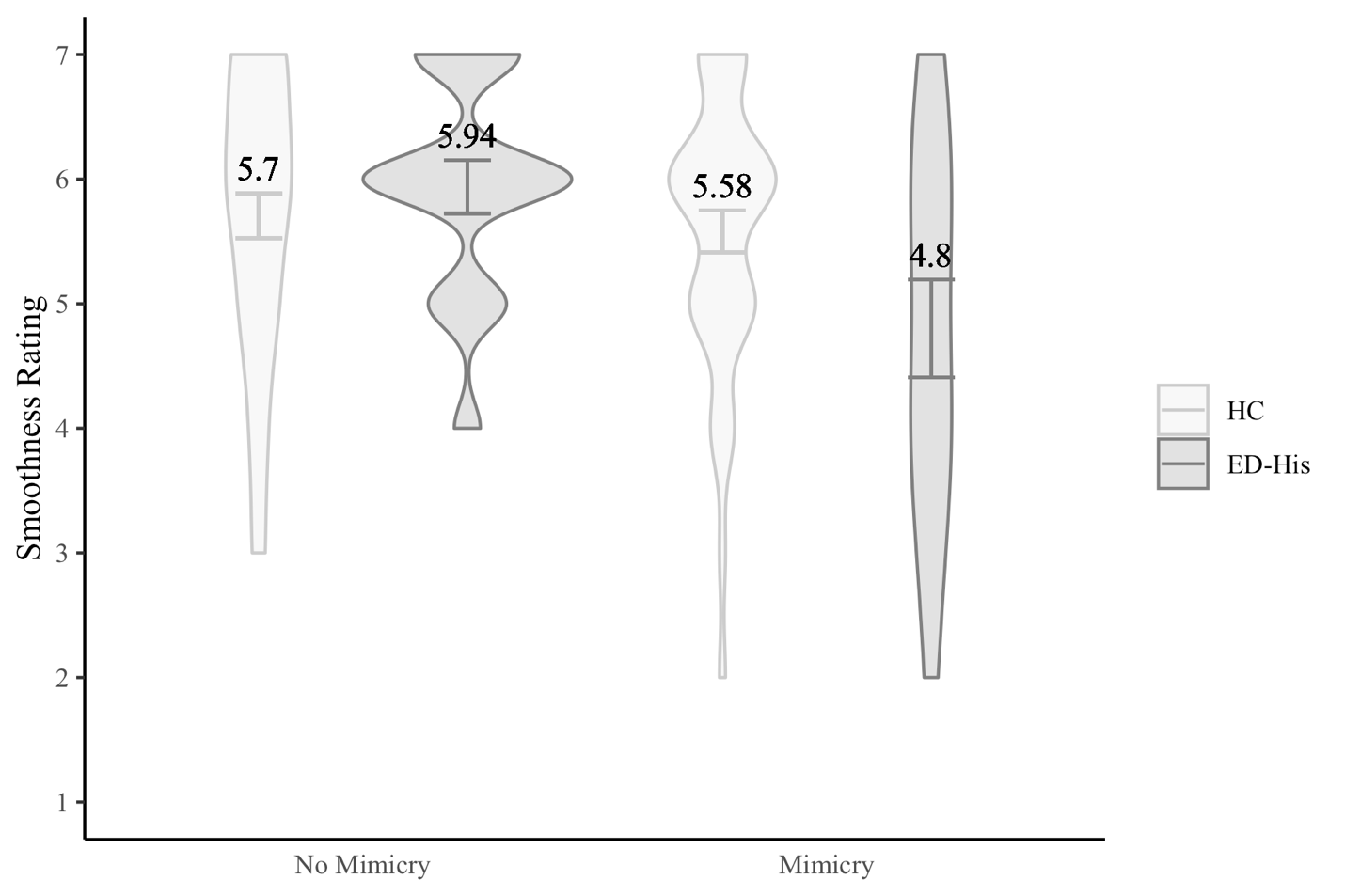


*Note*. Violin plots display the distribution of smoothness ratings. Participants rated the smoothness of the interaction with the confederate on a scale of 1 (extremely awkward) to 7 (extremely smoothly). Within the Mimicry condition, participants with a history of an eating disorder (ED-His) rated the interaction as less smooth than healthy control (HC) participants. There was not a significant group difference within the No-Mimicry condition. Error bars represent standard error.
